# Inhibition of Porcine Aminopeptidase M (pAMP) by the Pentapeptide Microginins

**DOI:** 10.3390/molecules24234369

**Published:** 2019-11-29

**Authors:** Glaucio Monteiro Ferreira, Thales Kronenberger, Éryka Costa de Almeida, Joseane Sampaio, Clélia Ferreira Terra, Ernani Pinto, Gustavo Henrique Goulart Trossini

**Affiliations:** 1 Department of Pharmacy, School of Pharmaceutical Sciences, University of São Paulo, Av Prof Lineu Prestes, 580, Bl. 13, São Paulo/SP CEP 05508-000, Brazil; gmf@usp.br; 2 Department of Oncology and Pneumology, Internal Medicine VIII, University Hospital Tübingen, Otfried-Müller-Straße 10, DE 72076 Tübingen, Germany; kronenberger7@gmail.com; 3 Department of Clinical and Toxicological Analyses, School of Pharmaceutical Sciences, University of São Paulo, Av Prof Lineu Prestes, 580, Bl. 17, São Paulo/SP CEP 05508-000, Brazil; erykaca@usp.br (É.C.d.A.); jsampaio@usp.br (J.S.); 4 Department of Biochemistry, Institute of Chemistry, University of São Paulo, Av Prof Lineu Prestes, 748, Bl. 12, São Paulo/SP CEP 05508-000, Brazil; clfterra@iq.usp.br

**Keywords:** aminopeptidase inhibition, cyanobacteria, Microcystis, microginin, molecular modelling

## Abstract

Aminopeptidase M (AMP) inhibition is of interest for several diseases, such as highly vascularized cancer types. AMP can be inhibited by linear pentapeptides isolated from *Microcystis aeruginosa* LTPNA08 (MG7XX). Porcine AMP inhibition—a model for human AMP—activity was spectrophotometrically measured by the formation of p-nitroanilide from L-leucine-p-nitroanilide substrate by AMP. AMP inhibition by MG770 exhibited comparable inhibition levels to amastatin (IC_50_ values: 1.20 ± 0.1 μM and 0.98 ± 0.1 μM, respectively), while MG756 was slightly less potent (with IC_50_ values of 3.26 ± 0.5 μM). Molecular modelling suggests a potential binding mode, based on the interaction with the Zn^2+^ cofactor, where MG770′s extra methyl group contributes to the disturbance of the Zn^2+^ cofactor complex and highlights the importance of hydrophobicity for the site.

## 1. Introduction

The aminopeptidase M (AMP, MEROPS number: M01.001) are extracellular anchored proteins, composed of a transmembrane domain which anchors it to the cell, and an ectodomain with approximately 930 amino acid residues, and heavily glycosylated [1,2]. AMP is commonly active as an anchored surface dimer, however, can also be found as a monomer and even as a freely soluble ectodomain [3]. 

**Figure 1:**
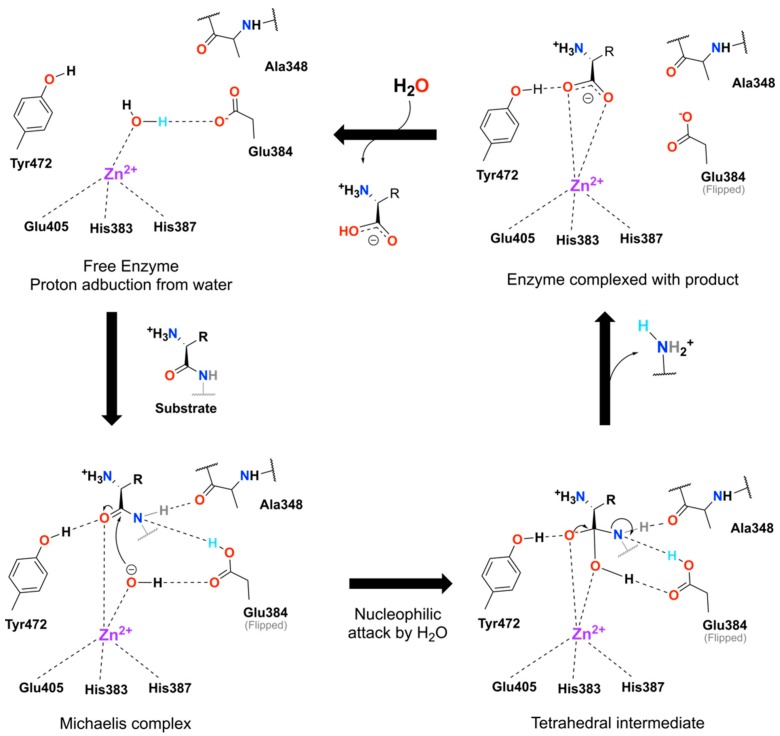
Aminopeptidase M (AMP) proposed catalytic mechanism. Starting from the free enzyme complexed with the structural water towards the activated state by a proton abduction from the water by the Glu384. Upon substrate binding the hydroxyl group nucleophilic attacks the carbonyl’s carbon in the Michaelis complex leading to the formation of the tetrahedral intermediate, which then reassembles itself expelling one aminated product. The cycle closes with the expulsion of the second product from the active site allowing the entry of a new water molecule. The ionized proton from the water is highlighted in cyan along the cycle. Mechanism was initially proposed by [6,7]. Dashed lines represent interactions and are for illustration purposes only, in the sense that angles and distances are not proportionally realists.

AMP ectodomain is composed of four sub-domains (I–IV) with the three *N*-terminal domains (I–III) being evolutionary conserved [4]. Particularly, sub-domain-II houses the highly conserved active site with the zinc-binding motif HEXXHX_18_ and GXMEN catalytic motifs, which has common mechanistic features with thermolysins and other peptidases [5]. Human and porcine AMP ectodomains share 79% of residue identity, together with the same domain architecture and dimerization pattern [2]. Human/porcine aminopeptidase proposed mechanism of action involves the peptide bond hydrolysis in two steps (illustrated in Figure 1 and based on the literature [6,7]). The first step involves proton abstraction from the water molecule, coordinated with the catalytic Zn^2+^ and stabilized Glu350 (not depicted in Figure 1), by the Glu384, which then allows the substrate entrance. The carbonyl oxygen of the substrate’s peptide bond is activated by zinc and Tyr472, while the alpha-nitrogen is stabilized by the Ala348 main-chain oxygen, in an intermediate state. As a last step, the peptide bond is hydrolyzed, by the catalytic water attack on the carbonyl carbon of the peptide bond, while the Glu384 shuttles a proton from the catalytic water to the leaving nitrogen group (Figure 1). Such a coordinated mechanism heavily relies in protonation state of individual residues, as shown by the work of Chen et al., 2014, where the porcine AMP loses activity in high pH solutions (likely due to the deprotonation of Tyr472) and in very low pH (due to the protonation of Glu384) [6,7].

**Figure 2:**
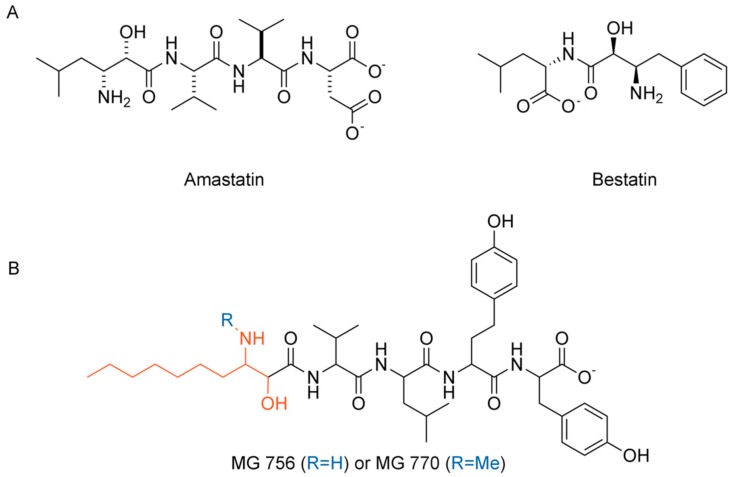
(**A**) AMP known inhibitors amastatin and bestatin. (**B**) Chemical structures of microginins MG 756 (R = H) and MG 770 (R = CH_3_), where the sequence is from the N- to C-terminal either 3-amino-2-hydroxydecanoic acid (Ahda in red) or N-Me-Ahda, valine, leucine, homotyrosine and tyrosine, and amastatin. Ahda stands for the modified amino acid 3-amino-(2-hydroxy)-decenoid acid.

Aminopeptidases have been extensively explored in recent years for a myriad of drug discovery applications [4]. Specifically, the relevance of AMP as a drug target against cancer has been supported by its role in promoting angiogenesis, tumor growth, and metastasis [8,9]. AMP overexpression on neovasculature of certain tumor cells, such as thyroid carcinomas and myeloid, also justify the drug discovery efforts [10,11,12] and, in this sense, aminopeptidase inhibitors have been considered potential targets for cancer therapy [13]. Furthermore, antitumor drugs, which bind to AMP site, are currently being evaluated in clinical trials [14,15]. Among the comprehensive list of AMP inhibitors (reviewed by Amin et al., 2018 [16]), amastatin (Figure 2A) figures as a slow-binding, competitive inhibitor, from which the hydroxyl group/carbonyl system contributes to interaction with Zn^2+^ ion and, therefore, complex stabilization mimicking the transition state and chelating the catalytic ion. Other peptide-mimetics, both synthetic and from natural sources can also be employed as AMP inhibitors.

Cyanobacteria species, such as *Microcystis aeroginosa*, are common phytoplankton species that can occur in several lakes and water reservoirs worldwide; they are able to produce peptides as secondary metabolites with distinct biological activities and toxicity properties and great pharmacological potential [1,2,17,18,19,20]. Recent investigations demonstrated that some of those linear peptides, the microginins, possess the ability to inhibit various proteases, including trypsin, chymotrypsin, angiotensin-converting enzyme (ACE) and some peptidases [4,5,13,21]. The main variants of microginins may contain from four to five amino acids and the presence of the unusual amino acid 3-amino-(2-hydroxy)-decenoid acid (Ahda) at N-terminus [4,5,13] (highlighted in red, Figure 2B).

Considering the ability of cyanobacteria to produce microginins, the present paper reports the isolation and characterization of the two active cyanopeptides from the methanolic extract of *Microcystin aeroginosa* LTPNA 08 named MG756 (Ahda-Val-Leu-HTy-Tyr, Figure 2B, where HTy is homotyrosine) and MG770 (N-Me-Ahda-Val-Leu-HTy-Tyr, Figure 2B) and their enzymatic inhibitory activities against porcine aminopeptidase M (EC 3.4.11.2) (AMP). Due to the high similarity between human and porcine, we herein propose the use of the latter as a model for studying human AMP activity. Complementarily, we further suggest a competitive potential binding mode using molecular modelling studies of these compounds on AMP binding site.

## 2. Results

### 2.1. Porcine and Human Aminopeptidase Have Similar Active Sites

**Figure 3:**
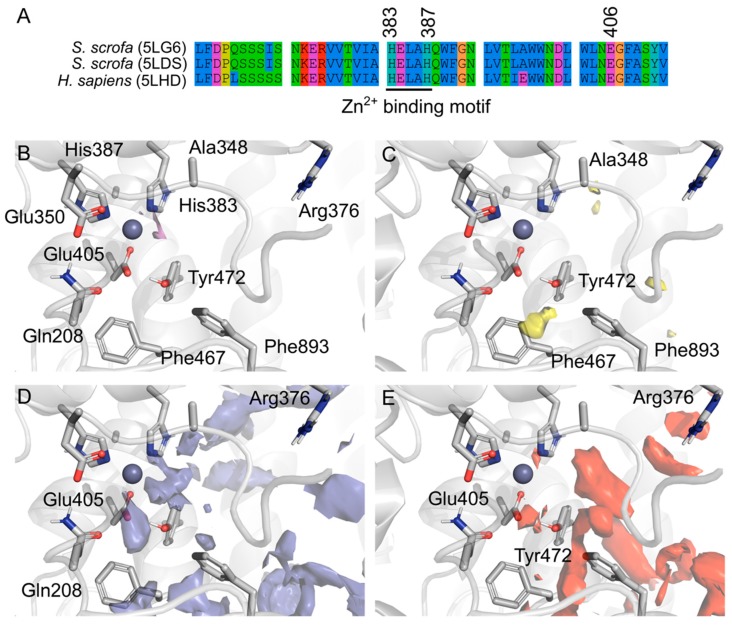
(**A**) Amino acid sequence alignment of Porcine AMP (PDB entries 5LG6 and 5LDS) and the human homologue (5LHD), overall human and porcine AMP share 79% of sequence identity and 87% similarity, however the binding domain is conserved with punctual changes. Amino acids are colored by property and the conserved Zn^2+^ binding motif is underlined. SiteMap prediction of the druggable binding site near the region of the amastatin binding site from the literature. Surfaces represent the different regions in the binding pocket and are colored by property: The metal coordination (purple, (**B**)), hydrophobic (yellow, (**C**)), hydrogen bond donors (blue, (**D**)) and hydrogen bond acceptors (red, (**E**)).

Porcine and human AMP amino-acid sequences were aligned and superimposed highlighting the high similarity between them and very conserved Zn^2+^ binding motif (Figure 3A), which is structurally supported by residues prone to metal complexation (Figure 3B). Overall, the active site pocket has a D-score of 1.16 on the SiteMap predictions (Schrödinger Inc, implemented in Maestro Drug Discovery Suite 2019), which is consistent with other validated drug targets [22]. AMP active site has several points likely to have hydrogen bond interactions (Figure 3D–E), which highlights the role of hydration within this active site. However, comparatively, there are less hydrophobic interactions (Figure 3C), mainly within a specific pocket composed by Ala346, Phe467 and Phe893. This back-pocket could be exploited for selectivity. Phe893 have been previously described as involved in the big conformational changes of AMP activation, from an open and receptive towards a closed and active conformation [1,2]. It was suggested that Phe893 would hinder the peptide release after the hydrolysis, despite the long distance apart from the active site (4.5 A^°^ away from the Zn^2+^).

### 2.2. Isolation and Identification of MG756 and MG770 from Microcystis aeroginosa LTPNA 08 and pAMP Inhibition

**Figure 4:**
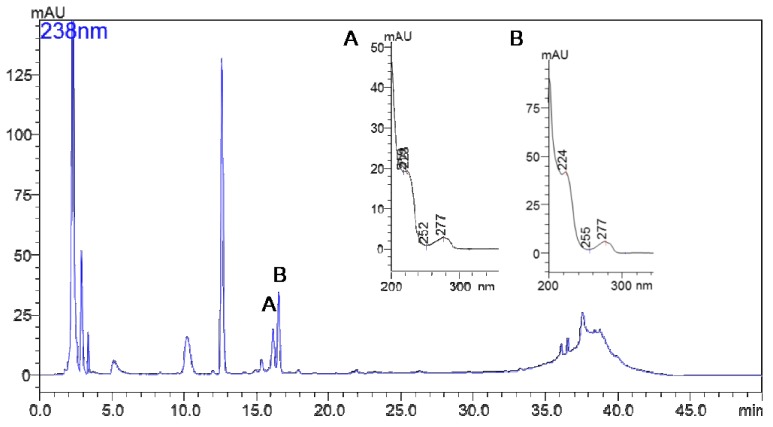
Chromatogram of LTPNA 08 strain by HPLC-DAD obtained by following as described in the methods section. (**A**) is the UV spectrum of peak at 16.1 min and (**B**) is the UV spectrum of peak at 16.8 min.

A typical chromatogram by HPLC-DAD of the strain LTPNA08 extract is shown in Figure 4. MG 756 and MG 770 eluted at 16.1 and 16.8 min, respectively. UV-VIS spectra of these two peaks are pointed as (A) and (B), showing a typical λ_max_ of microginins, as previously described by Carneiro et al., 2012 [23] and Paiva et al., 2017 [24].

**Figure 5:**
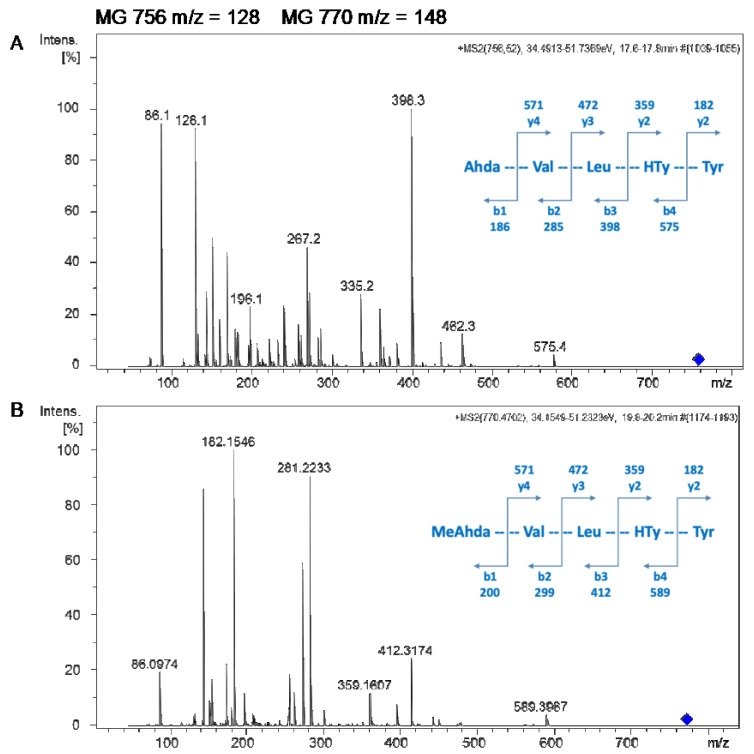
MS/MS spectra of MG 756 (**A**) and MG 770 (**B**). Ion attribution based on accurate mass, isotopic distribution and fragmentation pattern allowed the identification of the b and y ion series for both microginins (schemes are in blue).

Microginins MG 756 and MG 770 were further characterized by LC-MS-QTOF, based on high resolution, isotopic distribution, and annotation of product ion spectra of *m*/*z* 756.5 and *m*/*z* 770.5, as shown in Figure 5A,B, respectively. Both spectra contain ions at *m*/*z* 128 and *m*/*z* 142, which are typical losses of the dissociation between C2 and C3 in the N-terminal residue 3-amino-2-hydroxydecanoic acid or N-methyl-3-amino-2-hydroxydecanoic acid, respectively. The tentative characterization of microginins is Ahda-Val-Leu-HTy-Tyr (MG756, Figure 5A) and Me-Ahda-Val-Leu-HTy-Tyr (MG770, Figure 5B), where HTy stands for homo-tyrosine.

**Figure 6:**
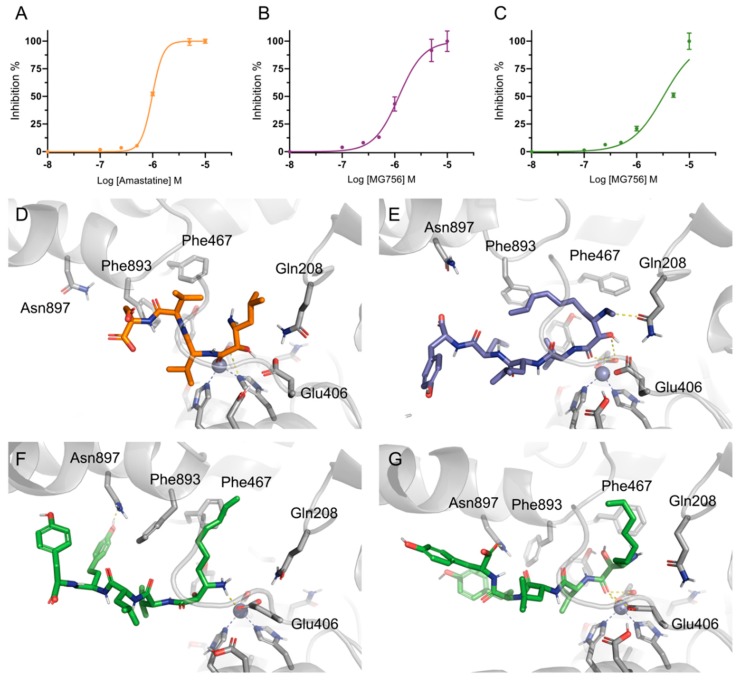
pAMP is inhibited by amastatin and microginins. Inhibitory curves (IC_50_ curves) of the Amastatin (**A**), MG770 (**B**), and MG756 (**C**); each line represents an individual independent experiment (R1–3). The proposed binding mode from a representative frame of the molecular dynamics’ simulations from AMP with the three compounds: Amastatin (**D**, in orange), MG770 (**E**, in purple), and MG756 (in green, with the amine protonated, **F**, and unionized, **G**).

MG756 and MG770 were purified from *Microcystis aeroginosa* LTPNA 08 and tested against porcine AMP purified protein. Both purified microginins had low micromolar inhibitory activity against pAMP, in similar range to amastatin (IC_50_ = 0.98 μM, Figure 6A, R^2^_avg_ = 0.99), where MG756 (Figure 6B, IC_50_ = 3.26 ± 0.5 μM, R^2^_avg_ = 0.94) was slightly less potent than MG770 (Figure 6C, IC_50_ = 1.20 ± 0.1 μM, R^2^_avg_ = 0.99).

### 2.3. Single Methylation Can Change the MG’s Binding Mode in the Porcine Aminopeptidase

Molecular docking studies were performed to investigate the possible binding mode of MG756 and MG770 within the active site of the porcine aminopeptidase. Amastatin ligand co-crystallized with the human aminopeptidase structure (PDB ID: 4FYT, hAMP) was used as the basis for cross-docking validation. The root means square deviation (RMSD) differences between the best-scored docking pose and the ligand coordinates from the crystal were employed as initial quality criteria (1.9 Å [25]). However, due to big conformational differences between the hAMP and pAMP, only cross-docking was not sufficient to evaluate docking quality. Therefore, the observation of important interactions, such as the distance towards the zinc ion (3.9 Å), further supported the use of docking, which was also analyzed by molecular dynamics simulations. The proposed binding mode involves hydrogen bond between amastatin’s hydroxyl group with the side-chain of the Glu406 residue, which is responsible for the Zn-binding domain along with His383 and His387. Based on the simulation results, we hypothesize that amastatin interaction with Glu450 could help with the initial orientation the coordination of the vicinal carbonyl/hydroxyl pair in a conformation that stably interacts the Glu406/His383/His387/Zn^2+^ complex.

**Figure 7:**
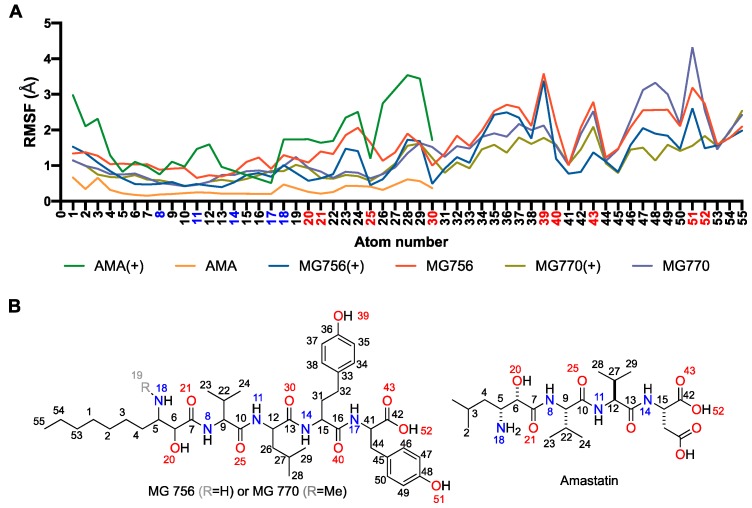
Microginins specific moieties, when compared to amastatin (green/orange lines), have highly flexible ligand binding within the binding pocket, as noted by the high root-mean-square deviation (RMSD) average along with the short simulation (**A**). Lines represent the average of RMSF values, which were calculated using ligand’s heavy atoms only, and compared against the initial conformation. Ligands with (+) represent the amino ionized form on atom 18. Atom numbers are described by the chemical 2D representations below (**B**), different ionization states are not represented in those figures. These results represent the interaction frequency along 500 ns of molecular dynamics simulation, as described in the methods section.

**Figure 8:**
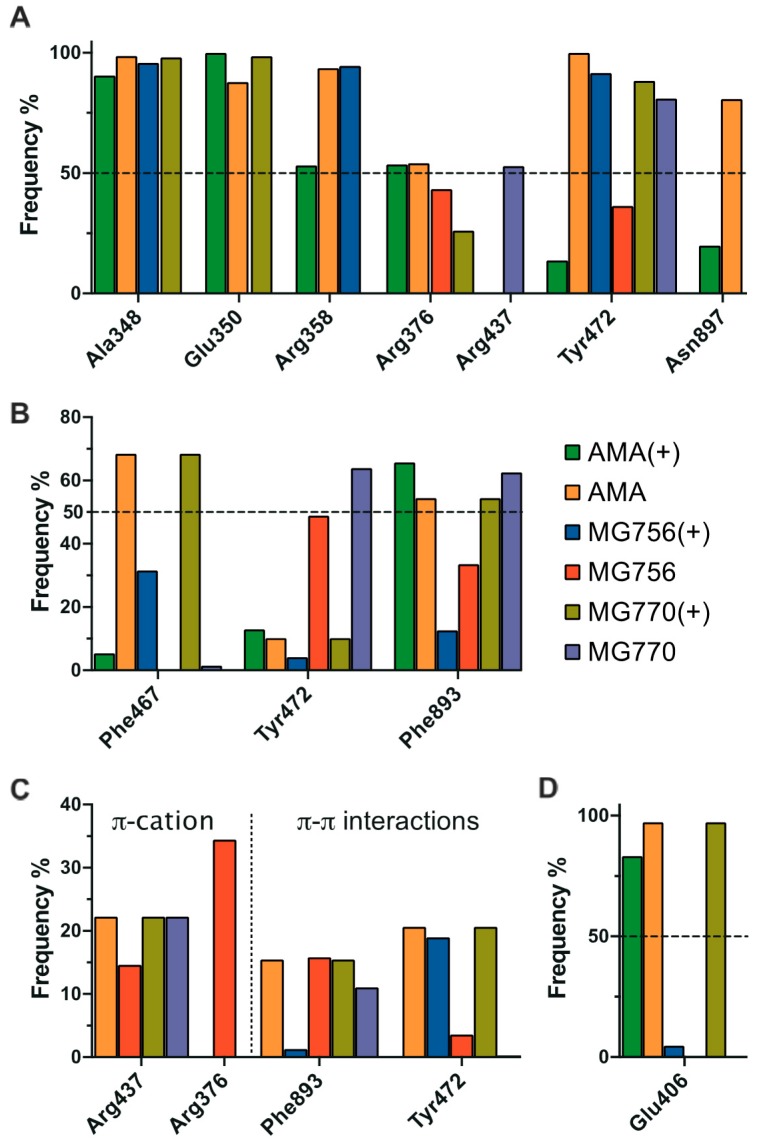
Interaction profile of amastatin (green bars represent ionized forms and yellow neutral forms) in comparison with MG756 (red for neutral amine state and blue bars representing the ionized amine) and MG770 (purple for neutral amine and dark yellow represented the ionized amine group), in terms of hydrogen bond (**A**), hydrophobic (**B**), pi-pi/pi-cation (**C**), and ionic interactions (**D**). These results represent the interaction frequency along 500 ns of molecular dynamics simulation, as described in the methods section.

The proposed binding mode of MG770 (Figure 6E) and MG756 (Figure 6F,G) have common features with the aforementioned amastatin binding mode (Figure 6D), such as the O21 carbonyl’s interaction with the Zn^2+^, which remains stable through the MD simulation (data not shown), however amastatin further interacts using the free hydroxyl, whereas the MG series does not. The twin tyrosine residues from microginins are longer and uncharged substituents to the double charged moieties of amastatin, it is observed that those residues are highly flexible and are not restricted to a single binding mode within the binding site (Figure 7A and Figure 8A, highlighting the hydrogen interaction profile), however, longer simulations would be required to understand the full effect of this in the protein dynamics. The crystal structure of hAPN (PDB ID 4FYS) co-crystallized with AngIV substrate peptide shows poorly fitting electron density and high-temperature factors for the last three amino acids (His-Pro-Phe residues and occupying the pockets P3′–P5′), as well as for the amastatin’s second amino-acid (LeußN, PBD 4FYT); those are in line with highly disordered regions and fit to our increasingly RMSF values along the molecule (Figure 7A). Nevertheless, previous structure-activity studies indicated that dityrosine C-terminus plays a key role in ACE inhibition [26].

The differences between activity between MG770 and MG756 could be attributed to the presence of a weaker interaction with the zinc ion for the ionized state of MG756. Simulations with the ionized amine state of MG756 shows increased ligand flexibility on Ahda atoms (Figure 7A), which would interact with the active site, but also overall binding instability. However, the same is not observed in MG770 ionized simulations. We hypothesize that the additional charge on MG756 could prevent the conjugation with Zn^2+^, despite the additional electrostatic interaction with the Glu450, which was not stable during simulations (Figure 8E). Furthermore, amastatin isopropyl moiety proximity with specific side-chains (Figure 6D) reveals a small unoccupied hydrophobic pocket composed by Phe467 and Phe893 (with distances larger than 5 Å), which accommodates the larger microginin’s acyl substituent (Figure 8B). Crystal structures of hAPN bound to AngIII–IV peptides show the substrate valine residue’s side-chain accommodated within this hydrophobic pocket [5]. Further, human APN crystal structure bound to bestatin shows PheßN residue’s hydrophobic moiety deeply inserted in this pocket, which highlights the potential flexibility of the region.

## 3. Discussion

Amastatin is long known to inhibit angiotensin-converting aminopeptidase enzyme (ACE) [26] and, while microginin-like peptides can inhibit both AMP and ACE, they are inactive against proteases such as trypsin, thrombin, plasmin, chymotrypsin, elastase, and papain (inhibitors were tested at 100 μg/mL) [27]. Our research group recently also demonstrated the mechanism of ACE’s inhibition by microginins [24], which we here propose would happen by a similar mechanism as amastatin/bestatin. Where the Ahda moiety would mimic the transition state and stabilize the enzyme in an inactive conformation.

However, despite the large biological potential, a comprehensive SAR would be necessary to improve selectivity and prepare this class to be used as new drugs in the therapy. Previous SARs showed that acetonide analogues, protecting the microginin’s free amine, lead to at least ninety times less activity against ACE [27]. The human AMP crystal structure co-crystallized with AngIII and AngIV peptides reveals a similar binding mode [5], where the Tyr477 (Tyr472 in pAMP) is proposed to stabilize the oxyanion generated in the transition state [28]. Additionally, the α-amino group of the Val residue in the peptide substrate have a hydrogen-bond interaction with the residues Glu355 (curiously, from the _352_GXMEN_356_ motif), Glu411 and Gln213 from the AMP protein.

Lastly, MG770 and MG756 are larger and more branched than amastatin (MG770 > MG756 > amastatin), which would allow them to occupy pockets amastatin cannot, such as Phe467, Tyr472, and Phe893. However, just molecular size cannot explain the differences in inhibition. Comparatively, the angiotensin-converting enzyme (ACE) inhibition, another member of the aminopeptidase family with the similar catalytic domain and zinc ion as a cofactor, shows an inverse correlation between molecular volume and activity [29,30,31]. As observed by the SiteMap analyses (Figure 3E), the closed conformation of the pAMP active site have small contributions of hydrophobic residues and, therefore, the insertion of a methyl group (increasing hydrophobicity), could be deleterious, when not addressing proper pockets. Controversially, the amino-ionized forms of MG770 maintained stable interactions with residues around the Zn^2+^ ion, whereas the MG756 equivalent does not. Recently, the work from Lodin-Friedman and Carmeli (2018) demonstrated the activity of other microginins, purified from *Microcystis* spp. collected from the Kishon Reservoir in Israel, against the porcine aminopeptidase M [32]. Those new microginins differ among themselves not only in terms of sequence, but also in absolute configuration of the Ahda moieties’ chiral centers, and also on the N-methylation of the Ahda amine group (Ahda stands for the modified amino acid 3-amino-(2-hydroxy)-decenoid acid). They conclude that Ahda-N-methylation, and even the chlorination of the Ahda terminal methyl group, did not influence the extent of the aminopeptidase M inhibition, which is in line with our observations that apolar groups would not lead to inactivity.

Inhibition of aminopeptidases is of pharmacological interest against highly vascularized cancer types. Microginin class of compound offers on-target activity with diverse modification points, however, has a large molecular weight, which can difficult further uses as new lead compounds. We here suggest an Ahda-dependent mechanism of action and, in this sense, one could use a reductionist approach to verify which fragments contribute to an increasing of binding affinity, without compromising the molecular core.

## 4. Materials and Methods

### 4.1. Growth and Cell Harvesting

Microcystis aeruginosa LTPNA 08 was cultivated in 10 L of ASM-1 medium bubbled with sterile air and grown at 22 ± 2 °C under 12 h light/12 h dark cycles (white fluorescent illumination—50 μmoL photon m^−2^ s^−1^) for 30 days. The culture was centrifuged at 3000 g for 5 min and the cells were lyophilized and used for further analysis.

### 4.2. Extraction, Isolation and Characterization of Microginins

MG 756 and MG 770 were extracted with the addition of 10 mL of methanol 90% in 50 mg of lyophilized cells of *Microcystis aeruginosa* LTPNA 08. Cyanobacterial cells were ruptured via probe sonication (amplitude of 30%, 2 min, Soni Omni Disruptor), centrifuged (9000× *g*, 4 C, 10 min) and concentrated under a stream of nitrogen. The material was kept under ultrasound (on an ice bath for 10 min) and samples were left on the bench for 1 h. After, the material was centrifuged at 3000 g, 4 °C for 20 min. The supernatants were collected and filtered through nylon filters (0.45 μm) using 1 mL syringes and packed in HPLC vials for chromatographic separation, following the methodology previously described by Paiva et al., 2017 [24]. Briefly, MG 756 and MG 770 were isolated in a semi-preparative scale (Luna C18 (2); 250 mm × 10 mm, 5 μm, Phenomenex^®^), using a Shimadzu Prominence HPLC system equipped with a LC-20AT quaternary pump and a SPDM20A photodiode array detector (DAD). Chromatograms were monitored at 276 nm λ_max_ and from 200 to 700 nm in the DAD detector. Suspected peaks were collected and analyzed by LC-HR-QTOF, as previously described by Carneiro et al., 2012 [23] and Paiva et al., 2017 [24].

### 4.3. Aminopeptidase M Inhibition

Inhibition assay spectrophotometrically performed using porcine aminopeptidase M (EC 3.4.11.2, from pig kidney—Sigma Aldrich^®^). After 30 min pre-incubation at 30 °C, 5 μL of the commercial aminopeptidase M enzyme (1 mU mL^−1^) added to microginin solutions (concentrations of 0.1, 0.25, 0.5, 1, 5 and 10 μM). For each concentration tested, the commercial enzyme was added and the positive control (inhibitor amastatin) was used for comparison (concentration: From 0.1 to 10 μM). The final volume at the 96-well plates was 75 μL (medium composed of 0.1 M borate buffer, Ph 8.0). After 30 min pre-incubation, 30 μL of the L-leucine-p-nitroanilide substrate at the concentration of 1.2 mM were added for the microginins AMP inhibition assays. The kinetics of inhibition of AMP were determined and the reaction interrupted with the addition of 25 μL of 30% acetic acid solution.

To obtain the factor used for the calculation of specific activity, a standard curve was performed with the product of the reaction (p-nitroanilide 1 mM), obtaining the slope of the curve. Absorbance reading was performed on the Spectra Max M2-Molecular Devices^®^ 96-well plate reader with SoftMax Pro^®^ software version 5.3, and the p-nitroanilide product concentrations were determined by the millimolar extinction coefficient at 405 nm as 9.96 (Sigma-Aldrich). After reading the samples, the residual activity calculations performed in mU mL^−1^, percentage of activity and inhibition. Activity curves were fitted to a non-linear regression model for normalized data using GraphPad Prism (v8.1, GraphPad Software, La Jolla, CA, USA). All assays were performed in triplicate and results represent the average of three independent experiments.

### 4.4. Molecular Modelling—System Preparation

The crystal structures of porcine aminopeptidase M were retrieved from the RCSB Protein Data Bank (PDB). Structure of the monomeric porcine (PDB ID: 5LDS, chain A) and human (PDB ID: 4FYT, chain A, used for comparison of the amastatin binding site) aminopeptidase N ectodomain was selected for studies (human and porcine AMP share 79% of sequence identity and 87% similarity). Since APN dimerization mediated by interactions between domain IV of both subunits is preserved among open, intermediate, and closed ectodomains [1], we decided to follow simulations with monomer structures. A single chain as a monomer was selected for each structure for molecular docking simulations, according to the number of Ramachandran outliers. The Small-Molecule Drug Discovery Suite (v2019-2, Schrödinger, LLC, New York, NY, 2019) was used for all calculations. Protein structures were prepared by adding hydrogen atoms and fixing missing side-chains using the Protein Preparation Wizard (PrepWiz, [33]) with default parameters, the zinc ion (Zn^2+^) was retained in the final structure. Ligands were drawn in Maestro and prepared by adjusting atomic charges and protonation states using LigPrep with default options, ligand protonation states were suggested by Epik, where the free amine of MG756 was suggested to exist both as ionized and neutral forms [34] (Supplementary material, Figure S1).

In parallel, SiteMap was used to evaluate the druggability of the binding pockets. This algorithm can predict the binding pockets in proteins based on the geometry, size, volume, and nature (hydrophilicity/hydrophobicity) of amino acid residues [22]. SiteMap can also express the druggability score of selected binding pockets in terms of D-score and visual analyses of the surfaces can provide further insights into the energetically favorable region for the binding mode of ligands.

### 4.5. Molecular Docking

The prepared protein structure was employed for docking studies, together with prepared ligands. In this sense, the initial conformation of MG770 was chosen based on the linear pentapeptide MeAhda-L-Val-L-Leu-L-HTy-L-Tyr, where MeAhda is the N-terminal was set as [2S,3R]-3-methylamino-2-hydroxy-decanoic-acid and HTy defines homo-tyrosine, the configuration of all other amino acids was based on previous reports [35,36,37,38]. Docking was performed using the GOLD (v5.6 [39]), where ligands were docked within a box of 15 Å radius around the enzyme’s active site, using the superimposed coordinates of amastatin, which contained the zinc ion. All residues within this pocket were considered flexible. Results derived from 30 independent rounds of genetic algorithm (GA) with a precision level of 100% and poses were ranked according to the GoldScore and CHEMPLP score values. The molecular docking results were visualized to verify the interaction of polar groups with the zinc ion and accommodation of the hydrophobic acyl groups. Poses were selected by Zn^2+^ interaction, however, due to the high diversity of potential binding modes, as well as poorly performing cross-docking, further validation was made necessary. In this sense, selected poses were then submitted to molecular dynamics simulation. PyMol software (v2.3.2, Schrödinger, Inc., New York, NY, USA) was used to produce all the images.

### 4.6. Molecular Dynamics Simulation

Molecular dynamics (MD) simulation was carried out using Desmond [40] with the OPLS3e force-field [41]. The simulated system encompassed the protein-ligand complexes, a predefined water model (TIP3P [42]) as a solvent and counterions (Na^+^ or Cl^−^ adjusted to neutralize the overall system charge). The system was treated in a cubic box with periodic boundary conditions specifying the shape and the size of the box as 13 Å distance from the box edges to any atom of the protein. We used a time step of 1 fs, the short-range coulombic interactions were treated using a cut-off value of 9.0 Å using the short-range method, while the smooth particle mesh Ewald method (PME) handled long-range coulombic interactions [43].

Initially, the relaxation of the system was performed using steepest descent and the limited-memory Broyden-Fletcher-Goldfarb-Shanno algorithms in a hybrid manner. The simulation was performed under the NPT ensemble for 5 ns implementing the Berendsen thermostat and barostat methods. A constant temperature of 310 K was maintained throughout the simulation using the Nose-Hoover thermostat algorithm and Martyna-Tobias-Klein Barostat algorithm to maintain 1 atm of pressure, respectively. After minimization and relaxation of the system, we proceeded with a single production step of 100 ns. The representative structure was selected by clustering the structures from the RSMD values, using 1 Å as a cut-off (Supplementary material, Figure S2 represents the variation of the RMSD values along with the simulation and the RMSF variation for the protein backbone, on Figure S3), small RMSF variation between the simulated systems was observed, this could be justified by the short simulated time, which allows for the observation of small time-scale events, but not big protein conformational changes. In this sense, simulations were used only to validate the initial docking results and evaluate the ligand stability within the protein active site. Interactions and distances were determined using the Simulation Event Analysis pipeline implemented in Maestro (Maestro 2019v2). The current geometric criteria for protein-ligand H-bond is distance of 2.5 Å between the donor and acceptor atoms (D—H···A); a donor angle of ≥120° between the donor-hydrogen-acceptor atoms (D—H···A); and an acceptor angle of ≥90° between the hydrogen-acceptor-bonded atom atoms (H···A—X). Similarly, protein-water or water-ligand H-bond are a distance of 2.8 Å between the donor and acceptor atoms (D—H···A); a donor angle of ≥110° between the donor-hydrogen-acceptor atoms (D—H···A); and an acceptor angle of ≥90° between the hydrogen-acceptor-bonded atom atoms (H···A—X). Non-specific hydrophobic interactions are defined by hydrophobic side-chain within 3.6 Å of a ligand’s aromatic or aliphatic carbons and π-π interactions required two aromatic groups stacked face-to-face or face-to-edge, within 4.5 Å of distance. Trajectories and interaction data are available on Zenodo repository (under the code: 10.5281/zenodo.3458133)

## 5. Conclusions

The results of the present study clearly indicate the aminopeptidase M inhibitory activity of microginins 756 and 770 on in vitro assays in the same range as the known inhibitor amastatin. Molecular modelling, by the means of docking and molecular dynamics analysis of the active site, suggests a binding mode where the extra methyl group of MG770 improves hydrophobicity near the Zn^2+^ interaction site. Microginin inhibitory activity could also be attributed to additional with another hydrophobic portion pocket composed by Phe467 and Phe893.

## Supplementary Materials

The following are available online at https://www.mdpi.com/1420-3049/24/23/4369/s1, Figure S1: Ionization state prediction for the microginins determined by pKa calculations with three independent methods: Quantum mechanical calculation (performed by Jaguar, in red), empirical fragment-based calculation (performed with Marvin, in blue) and the experimentally determined hydroxyl’ pKa value for Triclosan (in black (1), as reported by Tobe et al., 1978, [44]). Chemical differences between MG770 and MG756 are highlighted in red, while the acquired proton is blue. Figure S2: Root mean square deviation (RSMD) values of the protein backbone for the four complex structures monitored along the three individual 500 ns production phase of the MD simulations. Figure S3: (A) Average residue fluctuation of the ligand-free simulations agrees with the temperature fluctuations (B-factors, from PDB ID: 5LDS in black line) from the crystal structure, with exception of the region between 560–600, which comprises part of the back-pocket interaction motif. Referring to ligand bound systems, average residue fluctuations obtained from root mean square deviation fluctuations (RMSD) of the pAMP backbone atoms calculated in relation to the initial simulation frame of the amastatin (B, green for protonated and C, orange line for neutral form), MG756 ionized amine form (D, blue) and neutral amine form (E, red), MG770 ionized (F, dark yellow) and neutral counterpart (G, purple), in all figures, RMSF values are compared against the ones derived from the simulation without ligand (gray line).
