# Supplementary material for: Inhibition of Porcine Aminopeptidase M (pAMP) by the Pentapeptide Microginins
Source: Molecules. 2019 Nov 29;24(23):4369. doi: 10.3390/molecules24234369 (PMC6930480; doi:10.3390/molecules24234369)
Supplement: Supplementary file 1 [file molecules-24-04369-s001.pdf]

Supporting Information

A

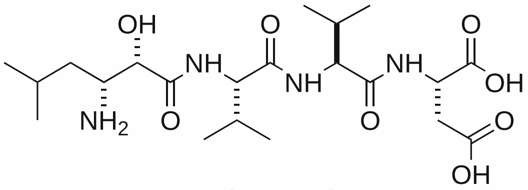

Amastatin

pKa = 8.0 [1]; pKa = 8.10; pKa = 7.90

B

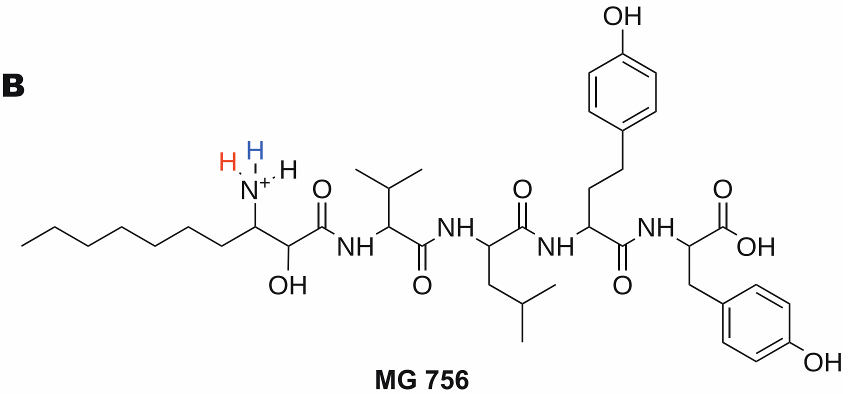

MG 756

pKa = 8.70; pKa = 11.80

C

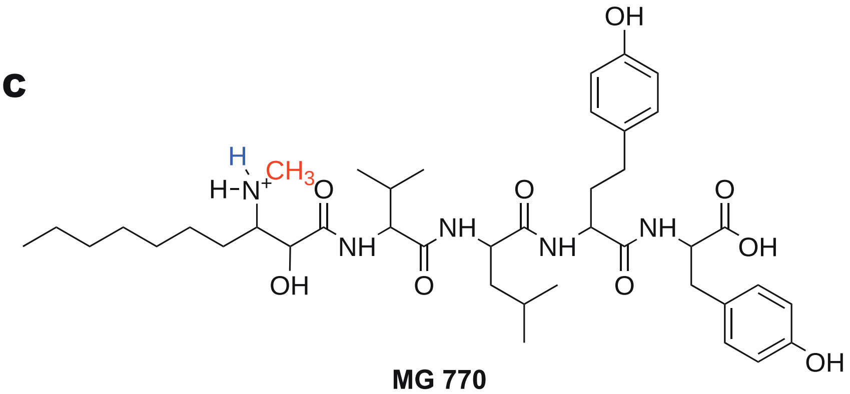

MG 770

pKa = 9.10; pKa = 11.80

Figure S1. Ionization state prediction for the microginins determined by pKa calculations for the free amino group. pKa calculation was performed with three independent methods: quantum mechanical calculation (performed by Jaguar, in red), empirical fragment-based calculation (performed with Marvin, in blue) and the experimentally determined hydroxyl' pKa value for Triclosan (in black (1), as reported by Tobe *et al.*, 1978, [40]). Chemical differences between MG770 and MG756 are highlighted in red, while the acquired proton is blue.

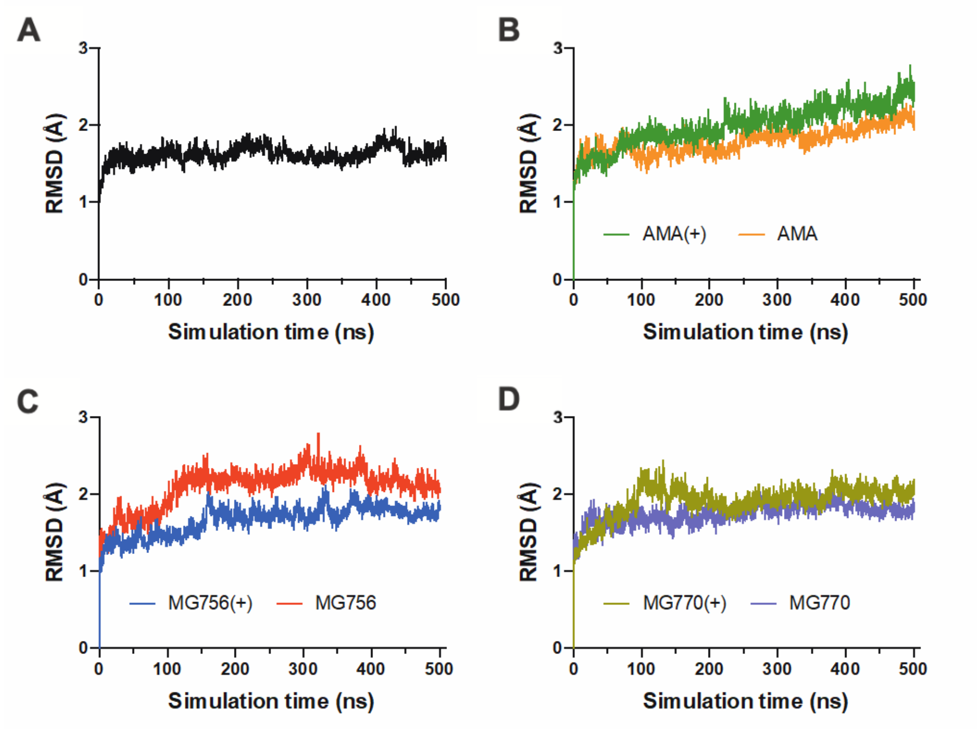

Figure S2. Root mean square deviation (RSMD) values of the protein backbone for the four complex structures monitored along the three individual 500 ns production phase of the MD simulations.

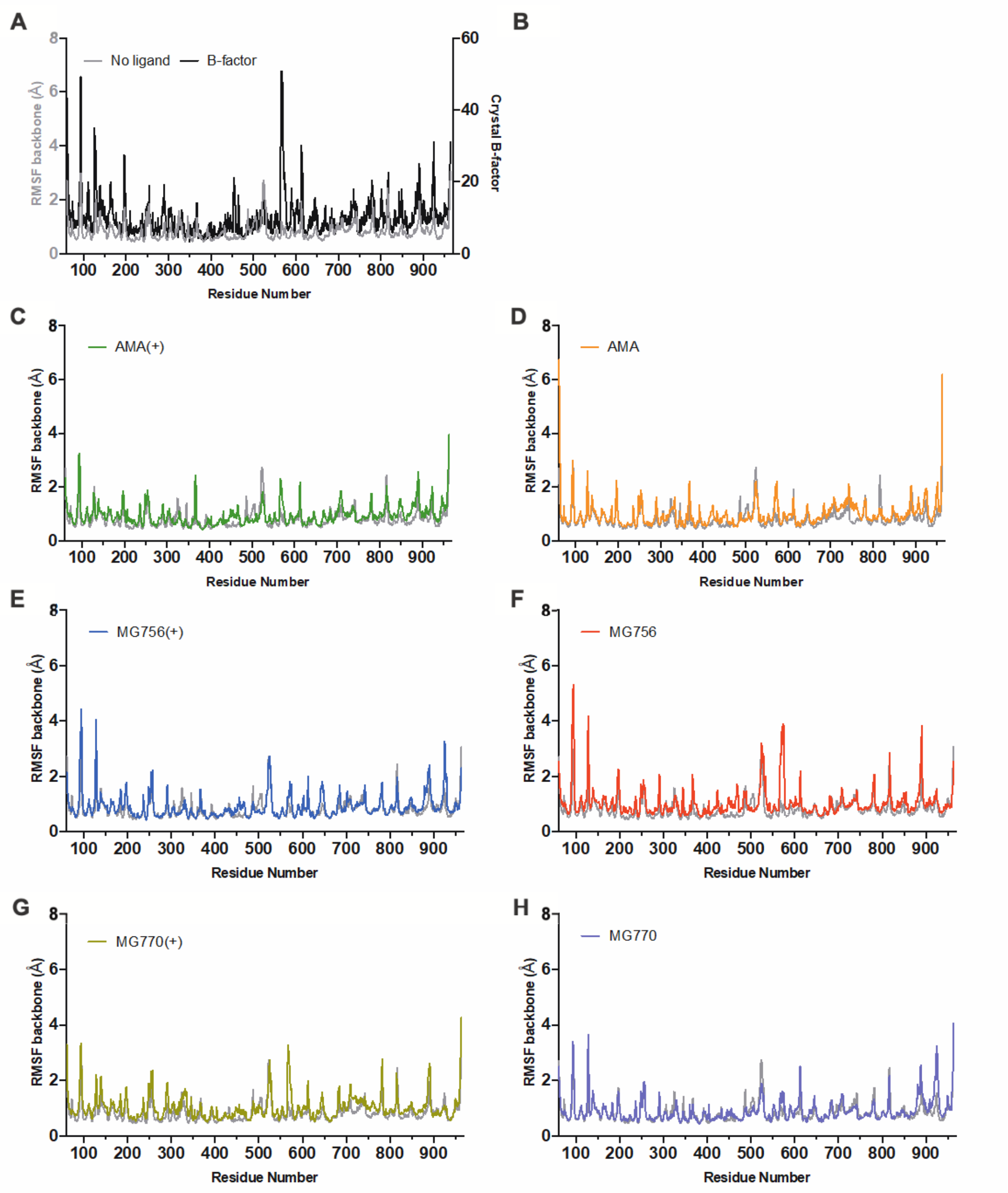

Figure S3. (**A**) Average residue fluctuation of the ligand-free simulations agrees with the temperature fluctuations (B-factors, from PDB ID: 5LDS in black line) from the crystal structure, with exception of the region between 560–600, which comprises part of the back-pocket interaction motif. Referring to ligand bound systems, average residue fluctuations obtained from root mean square deviation fluctuations (RMSD) of the pAMP backbone atoms calculated in relation to the initial simulation frame for Amastatin (**B**, green for protonated and **C**, orange line for neutral form), MG756 ionized amine form (**D**, blue) and neutral amine form (**E**, red), MG770 ionized (**F**, dark yellow) and neutral counterpart (**G**, purple), in all figures, RMSF values are compared against the ones derived from the simulation without ligand (gray line).
